# Author Correction: COVID-19 Disease Map, building a computational repository of SARS-CoV-2 virus-host interaction mechanisms

**DOI:** 10.1038/s41597-020-00589-w

**Published:** 2020-07-17

**Authors:** Marek Ostaszewski, Alexander Mazein, Marc E. Gillespie, Inna Kuperstein, Anna Niarakis, Henning Hermjakob, Alexander R. Pico, Egon L. Willighagen, Chris T. Evelo, Jan Hasenauer, Falk Schreiber, Andreas Dräger, Emek Demir, Olaf Wolkenhauer, Laura I. Furlong, Emmanuel Barillot, Joaquin Dopazo, Aurelio Orta-Resendiz, Francesco Messina, Alfonso Valencia, Akira Funahashi, Hiroaki Kitano, Charles Auffray, Rudi Balling, Reinhard Schneider

**Affiliations:** 1 Luxembourg Centre for Systems Biomedicine, University of Luxembourg, Belvaux, Luxembourg; 2 European Institute for Systems Biology and Medicine (EISBM), Vourles, France; 3 Ontario Institute for Cancer Research, Toronto, Canada; 4 College of Pharmacy and Health Sciences, St. John’s University, Queens, NY USA; 5 Institut Curie, PSL Research University, Mines Paris Tech, Inserm, Paris, France; 6 Department of Biology, Univ. Évry, University of Paris-Saclay, Genopole, 91025 Évry, France; 7 European Molecular Biology Laboratory, European Bioinformatics Institute (EMBL-EBI), Hinxton, UK; 8 Institute of Data Science and Biotechnology, Gladstone Institutes, San Francisco, United States; 9 Department of Bioinformatics-BiGCaT, NUTRIM, Maastricht University, Maastricht, The Netherlands; 10 Maastricht Centre for Systems Biology, Maastricht University, Maastricht, The Netherlands; 11 Helmholtz Zentrum München, Institute of Computational Biology, Neuherberg, Germany; 12 Center for Mathematics, Technische Universität München, Garching, Germany; 13 Faculty of Mathematics and Natural Sciences, University of Bonn, Bonn, Germany; 14 University of Konstanz, Department of Computer and Information Science, Konstanz, Germany; 15 Monash University, Faculty of Information Technology, Melbourne, Australia; 16 Computational Systems Biology of Infection and Antimicrobial-Resistant Pathogens, Institute for Bioinformatics and Medical Informatics (IBMI), University of Tübingen, 72076 Tübingen, Germany; 17 Department of Computer Science, University of Tübingen, 72076 Tübingen, Germany; 18 German Center for Infection Research (DZIF), partner site, Tübingen, Germany; 19 Department of Molecular and Medical Genetics, School of Medicine, Oregon Health & Science University, Portland, USA; 20 Department of Systems Biology & Bioinformatics, University of Rostock, Rostock, Germany; 21 Stellenbosch Institute of Advanced Study (STIAS), Wallenberg Research Centre at Stellenbosch University, 7602 Stellenbosch, South Africa; 22 Research Programme on Biomedical informatics, Hospital del Mar Medical Research Institute, Department of experimental and Health Sciences, Pompeu Fabra University, Barcelona, Spain; 23 Clinical Bioinformatics Area, Fundación Progreso y Salud. Hosp. Virgen del Rocío, Sevilla, Spain; 24 Bioinformatics in Rare Diseases. Centro de Investigación Biomédica en Red de Enfermedades Raras, Fundación Progreso y Salud, Hosp. Virgen del Rocío, Sevilla, Spain; 25 INB-ELIXIR-es, FPS, Hospital Virgen del Rocío, Sevilla, 42013 Spain; 26 Institute of Biomedicine of Seville (IBIS), Hospital Virgen del Rocio, 41013 Sevilla, Spain; 27 HIV, Inflammation and Persistence Unit, Virology Department, Institut Pasteur, Paris, France; 28 Bio Sorbonne Paris Cité, Université de Paris, Paris, France; 29 Dipartimento di Epidemiologia Ricerca Pre-Clinica e Diagnostica Avanzata, National Institute for Infectious Diseases “Lazzaro Spallanzani” i.R.c.c.S., Rome, Italy; 30 COVID 19 INMI Network Medicine for IDs Study Group, National Institute for Infectious Diseases “Lazzaro Spallanzani” I.R.C.C.S., Rome, Italy; 31 Barcelona Supercomputing Center (BSC), Barcelona, Spain; 32 Institucio Catalana de Recerca I Estudis Avançats (ICREA), Barcelona, Spain; 33 Department of Biosciences and Informatics, Keio University, Yokohama, Kanagawa Japan; 34 The Systems Biology Institute, Shinagawa, Tokyo Japan; 35 Okinawa Institute of Science and Technology Graduate University, Kunigami, Okinawa, Japan; 36 Sony Computer Science Laboratories, inc., Tokyo, Japan

Correction to: *Scientific Data* 10.1038/s41597-020-0477-8, published online 05 May 2020

Following publication, it was found that one of Alexander Mazein’s affiliations was missing and the affiliation, Barcelona Supercomputing Center (BSC) was incorrectly spelled.

It was also found that a part of the Acknowledgements section and a part of the Competing Interests section were missing.

Both the HTML and PDF versions have been updated to reflect these changes.

